# Medical Lectures and Clinical Aphorisms

**Published:** 1917-07

**Authors:** 


### Reviews of Books.

---

**Medical Lectures and Clinical Aphorisms.** By SAMUEL GEE, M.D., with Recollections by J. WICKHAM LEGG. Pp. viii. 392. Oxford Medical Publications. 1915. Price 6s. net.

In this work we have what in a former generation would have been termed "the remains" of a clinical observer and teacher, who was certainly not surpassed in the fields of observation and teaching by any one of his many distinguished contemporaries. All his writings, which are unfortunately few, are characterised by sound judgment, learning, and breadth of view. From them we can gather somewhat of the privilege it must have been to have been taught clinical medicine by such a master of the art. His writings show that his teaching was of the kind that provokes thought, and stimulates his hearers to further investigation.

This book consists of several lectures, his clinical aphorisms, and three extremely interesting essays on "Sects in Medicine," "The Conflict of Medicine with Small Pox," and on "Abraham Cowley." Dr. Wickham Legg adds his Recollections, which practically amount to a short biography and appreciation of the author, and helps the reader greatly in understanding what manner of man he was.

Of these various parts we take Dr. Wickham Legg's biographical sketch and the Clinical Aphorisms as likely to be of most permanent value. The one gives us a picture of a remarkable man of whom posterity will certainly want to know all that it can, and the other is the essence of the observations of one of the most acute observers at a great period in medicine. In reading these aphorisms we are struck with several instances in which the statements made are now common knowledge, and others in which Gee anticipated, and has been confirmed by later investigations, carried out by methods then unknown. It is given to few to be able to write aphorisms which stand the test of time and change; we think that the majority of these will do so. In conclusion, this is a book which every lover of medicine should have on his shelves, and we should place it side by side with that too little known work of another distinguished physician, a little antecedent to Gee, Moxon's *Pilocereus Senilis*.

